# Supplementary material for: Super‐Droplet‐Repellent Carbon‐Based Printable Perovskite Solar Cells
Source: Adv Sci (Weinh). 2024 May 2;11(26):2401016. doi: 10.1002/advs.202401016 (PMC11234403; doi:10.1002/advs.202401016)
Supplement: Supplementary file 1 — Supporting Information [file ADVS-11-2401016-s009.pdf]

ADVANCED SCIENCE
Open Access

Supporting Information

for *Adv. Sci.*, DOI 10.1002/advs.202401016

Super-Droplet-Repellent Carbon-Based Printable Perovskite Solar Cells

Cuc Thi Kim Mai, Janne Halme*, Heikki A. Nurmi, Aldeliane M. da Silva, Gabriela S. Lorite, David Martineau, Stéphanie Narbey, Naeimeh Mozaffari, Robin H. A. Ras, Syed Ghufran Hashmi and Maja Vuckovac**

Supporting Information

Super-Droplet-Repellent Carbon-Based Printable Perovskite Solar Cells

Cuc Thi Kim Mai, Janne Halme*, Heikki A. Nurmi, Aldeliane M. da Silva, Gabriela S. Lorite, David Martineau, Stéphanie Narbey, Naeimeh Mozaffari, Robin H. A. Ras, Syed Ghufran Hashmi and, Maja Vuckovac**

**Corresponding author email: Thi.Mai@oulu.fi, janne.halme@aalto.fi, maja.vuckovac@aalto.fi*

This PDF file includes:

- Supplementary Text
  - "Wetting background important for carbon-based printable perovskite solar cells"
  - "Encapsulated carbon-based printable perovskite solar cells"
  - "Oscillating droplet tribometer for wetting characterization"
- Supplementary Figures 1-13
- Supplementary Tables 1-14
- Description of Supplementary Videos 1-8
- Supplementary Materials References

Supplementary Text

Wetting background important for carbon-based printable perovskite solar cells

Here, we discuss the concept of hydrophobicity in the passivation process[1] introduced among other moisture barrier layers (Table S1). Due to the low surface energy, *hydrophobic* coatings reduce the contact area between water droplets and solid surfaces. In other words, low surface energy restricts the spreading of the droplets, resulting in droplets forming hemispherical or spherical shapes with contact angles between 90˚ and 150˚ ($90^\circ < \theta < 150^\circ$).[2] However, droplets on such coatings could experience significant friction and adhesion forces, resulting in sticky or immobile droplets.[3] Such droplets, over time, can damage the hydrophobic coating, allowing moisture to penetrate and further degrade PSCs (serves as a wetting defect, and water has a higher affinity to accumulate at the defect). This can be addressed by reducing contact points between droplet and surface using *superhydrophobic* (water super-repellent) coatings that combine roughness and low surface energy to achieve advancing contact angle $\theta_{\mathrm{adv}} > 150^\circ$ and contact angle hysteresis CAH < 10 ˚.[2] The CAH should be essential in PSC applications as it measures droplet mobility and how droplets advance on and recede from the surface. It is usually calculated as the difference between the advancing contact angle (θ_{adv}, measured by increasing droplet volume) and receding contact angle (θ_{rec}, measured by decreasing droplet volume). As the smaller CAH is, the droplet is more mobile[3] and will spend less time in contact with the surface, drastically reducing the chance for PSC moisture-induced degradation.

In general, for perovskite-based solar cells, the duration of droplet contact with perovskite is crucial due to their high sensitivity to moisture. Therefore, when employing repellent coatings, achieving high advancing and receding contact angles (>150˚) and small contact angle hysteresis (<10°) is essential. Consequently, accurate measurement and reporting of advancing contact angle, receding contact angle, and contact angle hysteresis are critical for assessing the effectiveness of superhydrophobic (water super-repellent) coatings. The research on perovskites only reports static contact angles that are shown to be an inadequate way for wetting characterization[4,5] because they do not account for droplet mobility or contact times. Thus, current studies (Table S1) employ rather hydrophobic than superhydrophobic materials[6–14] and usually a couple of hydrophobic (e.g., PDMS) barrier layers similar to sealant materials (dense and not only as the top layer)[11] to demonstrate improved stability of PSCs.

Encapsulated carbon-based printable perovskite solar cells

Pre-laminated glass lids covered with thermoplastic (Product Code: 76864, Solaronix) were first aligned on the CPSCs' carbon electrode, then carefully placed on a preheated (100 °C)

hotplate. As the thermoplastic melted, the lids were strongly fused to the non-active area of the cells. After the cells were cooled down to RT, copper tapes were attached to the edges of the CPSCs to create the contacts, which were then completed with quick-drying silver conductive paint (Electrolube, UK). Finally, the contacts and device edges were sealed with an epoxy sealant (Power Epoxy Extra Time 60 min, LOCTITE-USA), and the CPSCs were left overnight in the air to dry the epoxy.

Oscillating droplet tribometer for wetting characterization

Oscillating droplet tribometer (ODT) measurements were done to characterize superhydrophobicity of the super-repellent CPSC. In ODT, a dilute ferrofluid (0.2 vol.% nanoparticles) droplet oscillates in a harmonic potential well with zero vertical component created by two permanent magnets to measure the visous and friction force between the droplet and the superhydrophobic surface.[15,16] The motion of the droplet can be described as a harmonic oscillator with viscous and friction force with an analytical solution in the case without driving force.[17] The oscillation of the ferrofluid droplet was caused by moving the magnets in a sinusoidal fashion for two oscillations near the resonance frequency (around 5 Hz) of the droplet. The motion of the droplet was captured with a Phantom V1610 high-speed camera, and the location of the droplet was measured as its centroid. The location was fitted in the analytical solution of the harmonic oscillator equation to obtain the viscous and friction force. As the friction force measured with ODT cannot be directly compared to contact angle measurements done with contact angle goniometer, the contact angle hysteresis ($\theta_{\mathrm{hyst}} = \theta_{\mathrm{adv}} - \theta_{\mathrm{rec}}$) was calculated from the friction force measured with ODT. The friction force of the droplet sliding on a tiled plane can be described using Equation 1.[18]

$$F_{\mathrm{\mu}} = \frac{24}{\pi^3} D\gamma(\cos\theta_{\mathrm{rec}} - \cos\theta_{\mathrm{adv}}) \tag{1}$$

where F_μ is the friction force, D baseline width of the 2D droplet silhouette, γ is the surface tension of the liquid, θ_{rec} and θ_{adv} are the receding and advancing contact angles. Assuming that the friction force of the sliding droplet on a tilted plane and in ODT are the same, the contact angle hysteresis can be calculated based on Eq. 1. This allows comparing the friction measurements of ODT to the contact angle measurements of the contact angle goniometer. The hysteresis based on the ODT measurements corresponds to a contact angle hysteresis of 2.8° when assuming an advancing angle of 165°, the water-like ferrofluid surface tension of 72 mN/m[15], and measured baseline width of 0.90 ± 0.07 mm.

Supplementary Figures

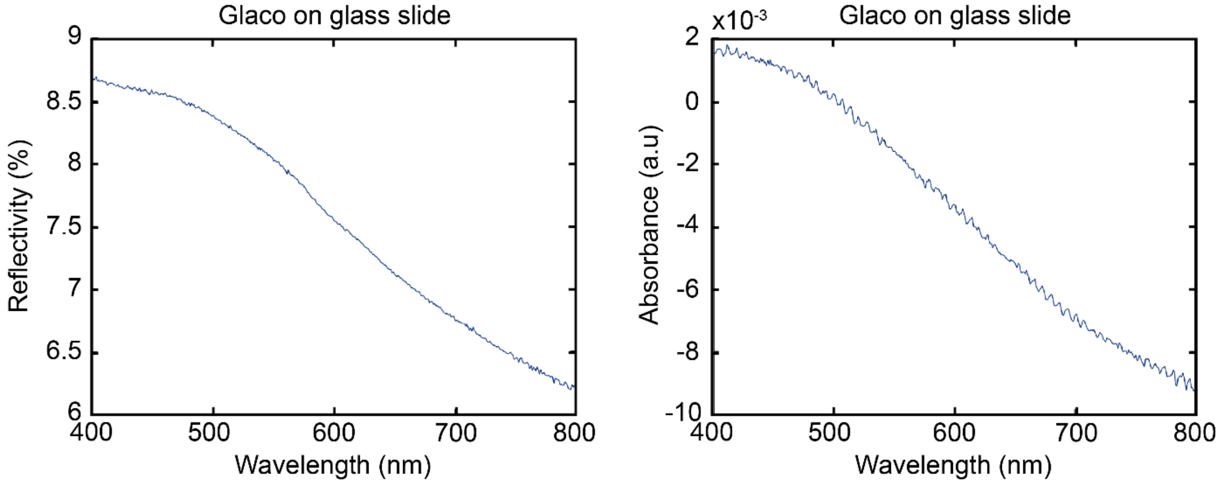

Figure S1. Reflectance and absorbance spectra of Glaco coating on glass slide.

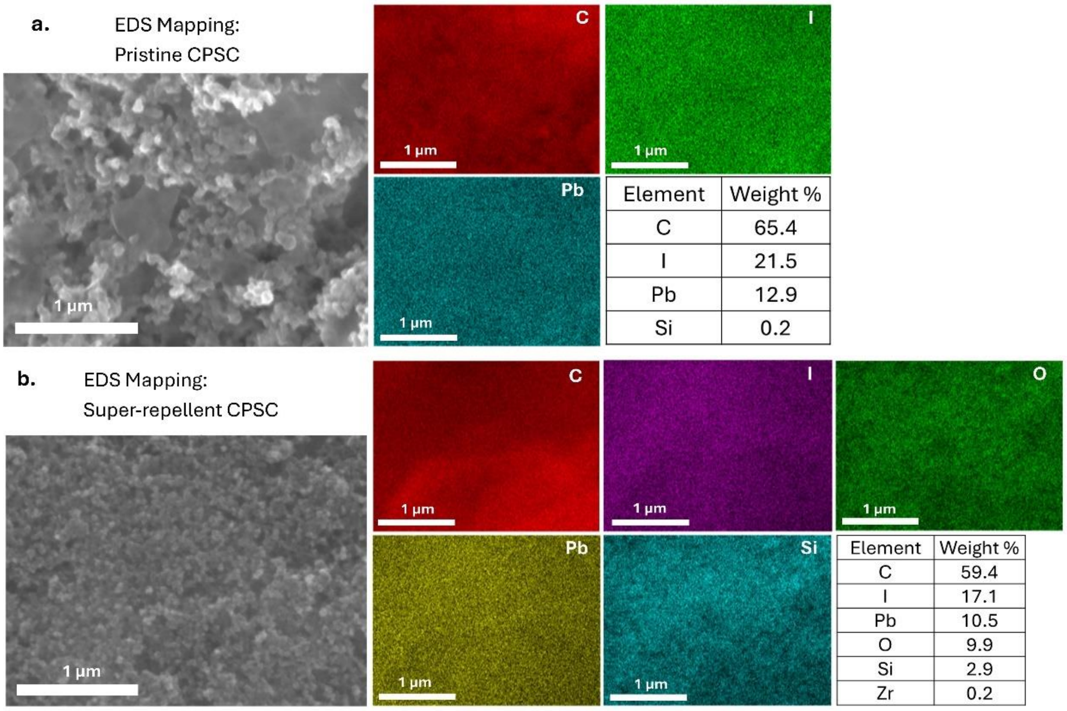

Figure S2. EDS mapping of composition of pristine CPSC (a) and super-repellent CPSC (b)

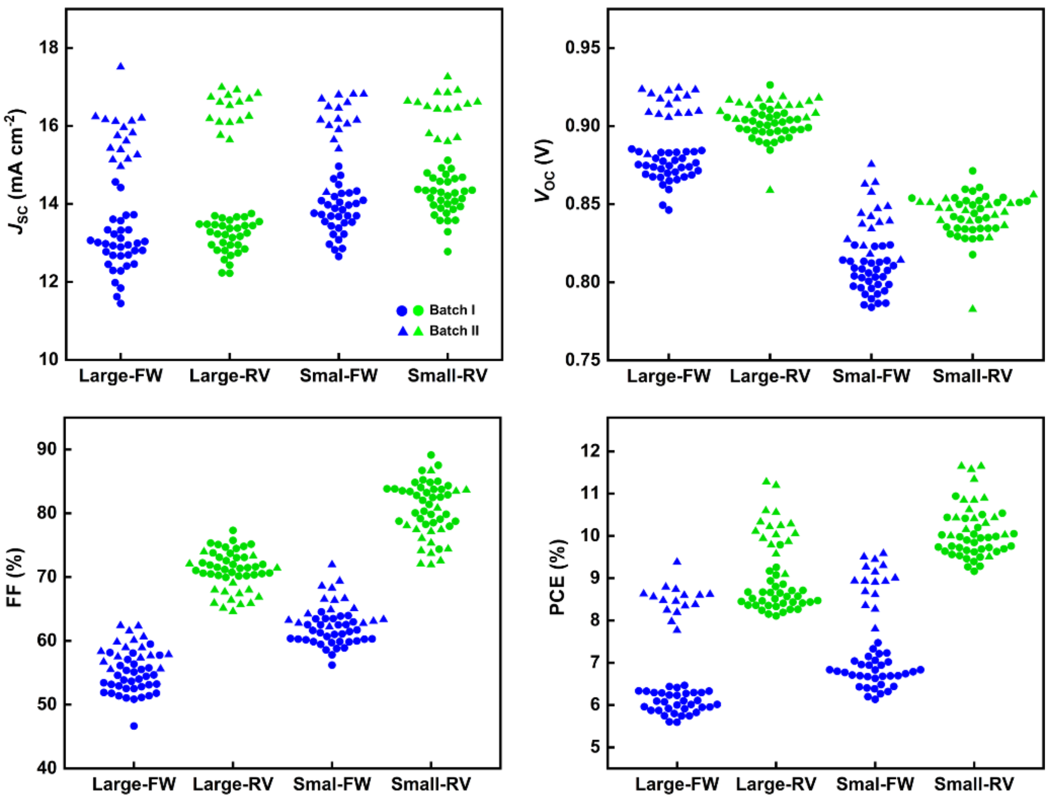

Figure S3. Initial PV parameters distribution (Forward - FW and Reverse – RV scans) of 50 unencapsulated CPSCs used in this study under 1 Sun condition. Among them, 34 CPSCs were from Batch I, 16 CPSCs were from Batch II. The CPSCs were measured with two mask-aperture areas: Large (0.64 cm^2) and Small (0.14 cm^2).

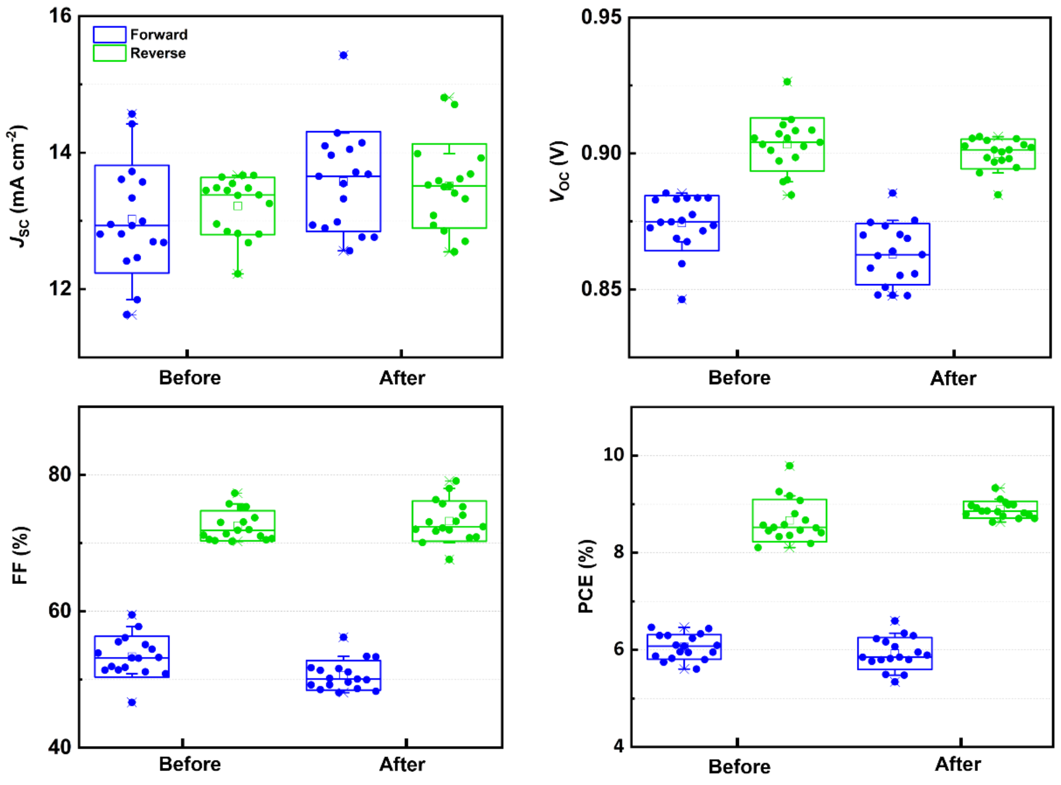

Figure S4. PV parameters distribution of 17 unencapsulated CPSCs from Batch I before and after super-repellent coating under 1 Sun condition. The measured aperture area was 0.64 cm^2.

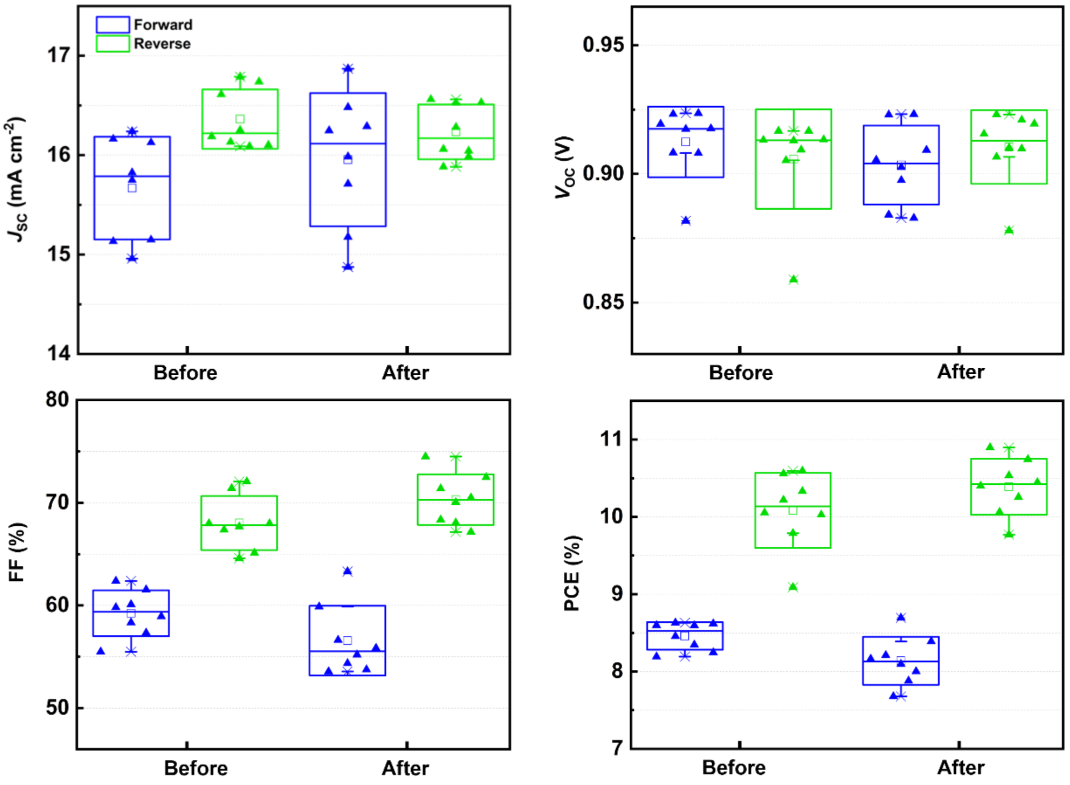

Figure S5. PV parameters distribution of 8 unencapsulated CPSCs from Batch II before and after super-repellent coating under 1 Sun condition. The measured aperture area was 0.64 cm^2.

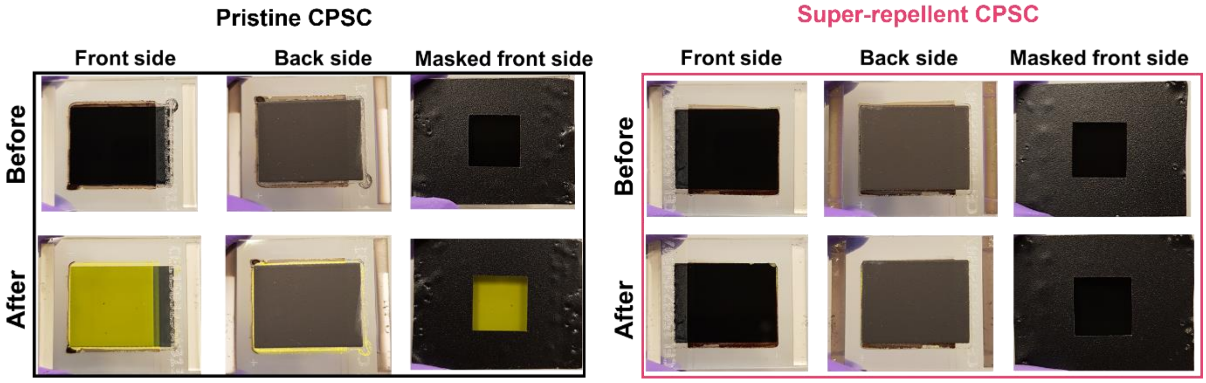

Figure S6. Photographic images of the pristine and super-repellent CPSCs before and after water dropping test.

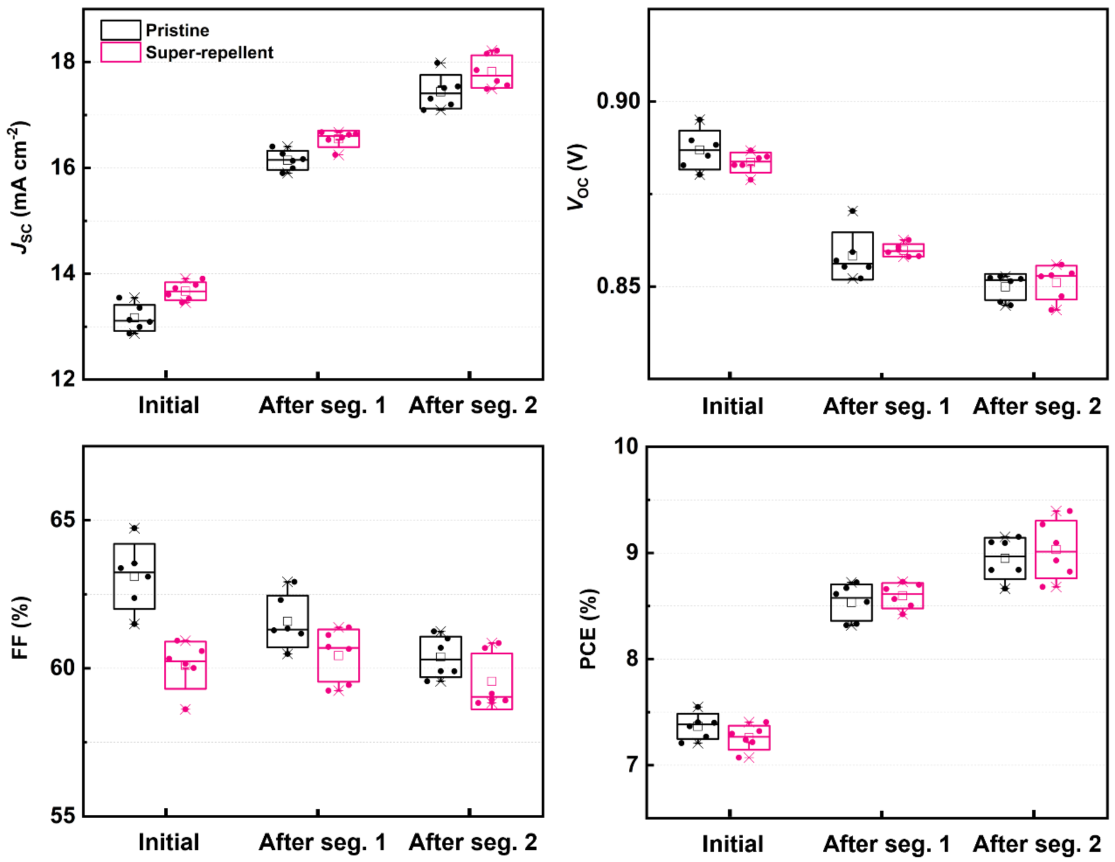

Figure S7. Average (of forward and reverse scans) PV parameters distribution of unencapsulated pristine and super-repellent CPSCs (6 cells in each group) under 1 Sun condition at the initial and after each segment of the dark storage aging test at room temperature (RT) and relative humidity (RH) 65% and 85%. The test consists of two segments: segment 1 was conducted at RT and 65% RH for 864 hours, and segment 2 was conducted at RT, 85% RH for 165 hours. The measured aperture area was 0.64 cm^2.

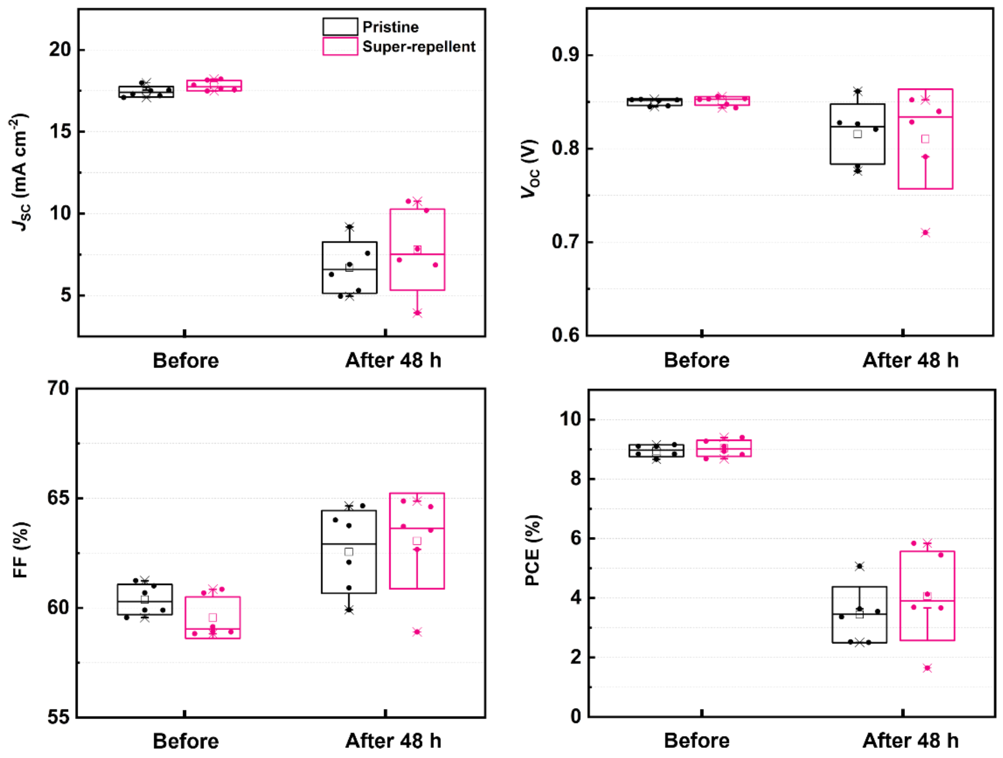

Figure S8. Average (of forward and reverse scans) PV parameters distribution of unencapsulated pristine and super-repellent CPSCs (6 cells in each group) under 1 Sun condition before and after the aging test at elevated temperature (40 °C). The measured aperture area was 0.64 cm^2.

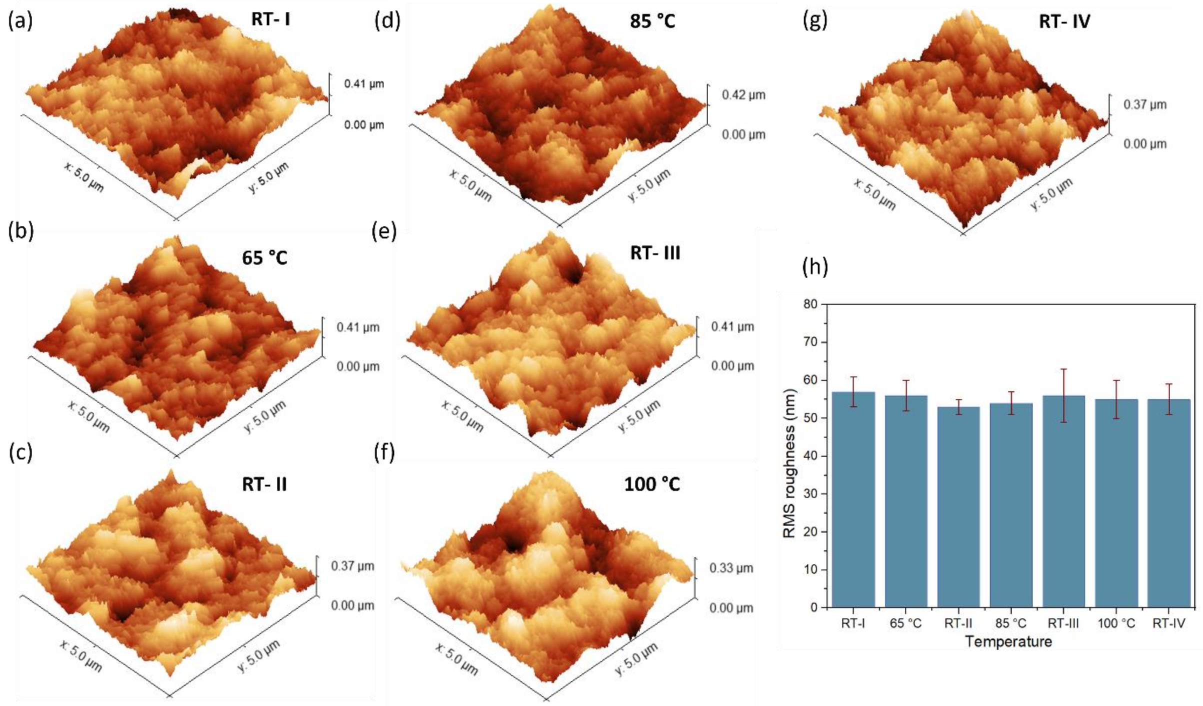

Figure S9. Representative AFM topography images (3D view) acquired for the same sample at different temperatures: (a) RT-I, (b) 65 °C, (c) RT-II, (d) 85 °C, (e) RT-III, (f) 100 °C, and (g) RT-IV. (h) RMS roughness averaged from 5 images at each temperature. The surface morphology looks similar for all measured temperatures and the RMS roughness does not present significant differences, indicating that the surface is stable under heating.

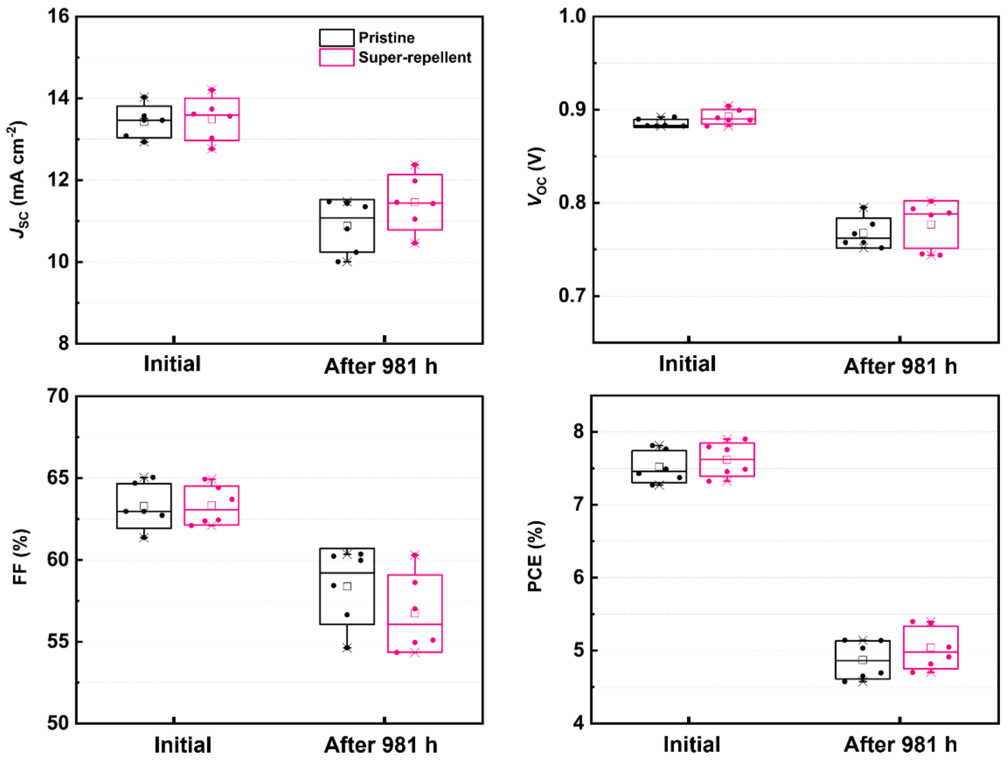

Figure S10. Average (of forward and reverse scans) PV parameters distribution of encapsulated pristine and super-repellent CPSCs (6 cells in each group) under 1 Sun condition at the initial and after the dark storage test at 65°C in ambient. The measured aperture area was 0.64 cm^2.

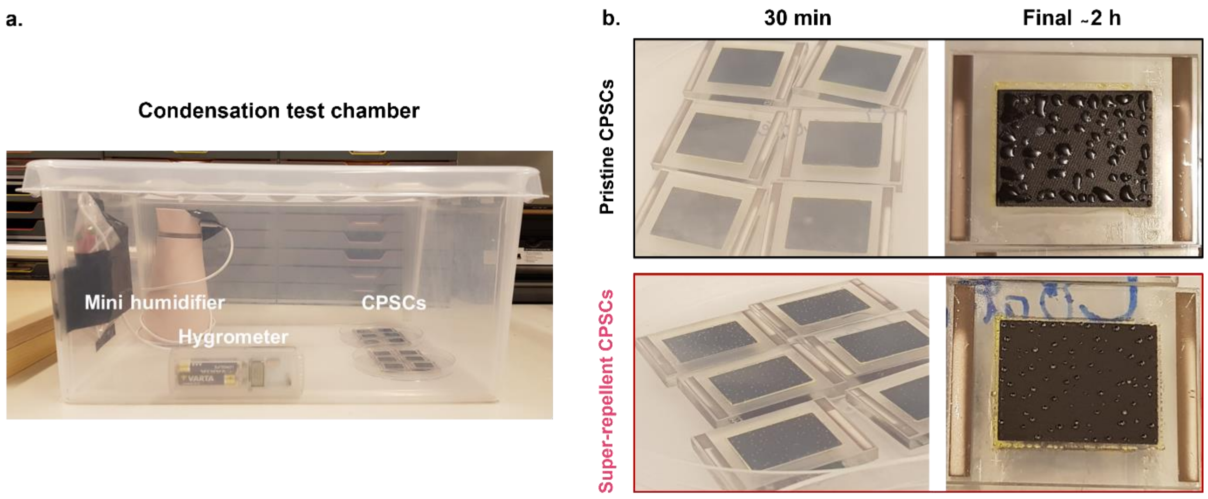

Figure S11. (a) Photographic image of homemade humidity chamber used for condensation tests (b) Photographic images of water droplets formed on pristine and super-repellent CPSCs after approximately 30 minutes of the condensation test (observing from outside of the chamber) and after the condensation test (~ 2 h).

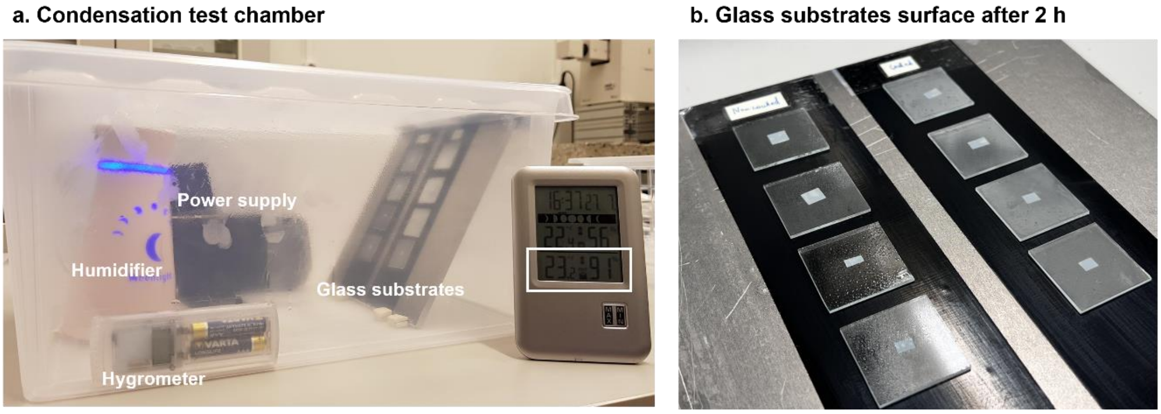

Figure S12. (a) Photographic image of homemade humidity chamber used for the condensation test where the pristine and super-repellent glass substrates were placed in a tilted position (b) Photographic images of water droplets formed on pristine (left side) and super-repellent (right side) glass substrates after 2 h of the condensation test.

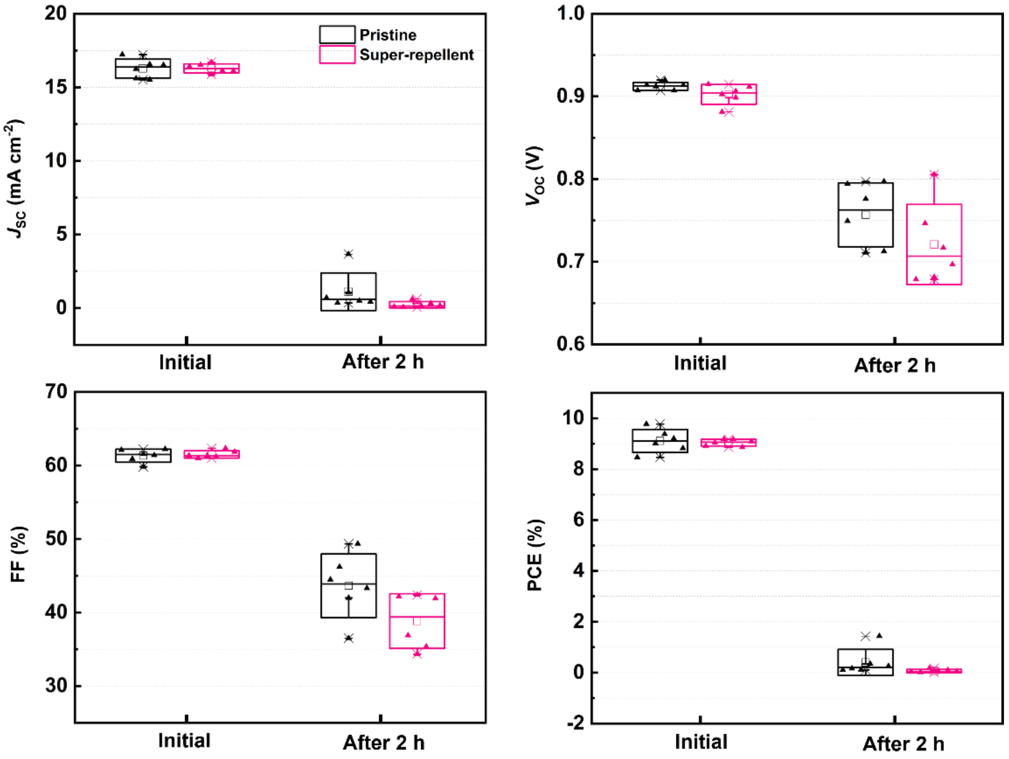

Figure S13. Average (of forward and reverse scans) PV parameters distribution of unencapsulated pristine and super-repellent CPSCs (6 cells in each group) under 1 Sun condition at the initial and after the condensation test. The measured aperture area was 0.64 cm^2.

Supplementary Tables

Table S1. Summary of hydrophobic materials integrated into perovskite (PVK) solar cells (PSCs). It is worth to note that most the reported contact angles are static, but advancing and receding contact angles (dynamic contact angle) have never been reported

PSC structure	Hydrophobic material	Deposition method	Contact angle (º)	Stability	Ref.
Glass/FTO/TiO_2/$MAPbI_3$/(CF_3-PEA)$_2$$PbI_4$/Spiro-OMeTAD/Au	2-D (CF_3-PEA)$_2$$PbI_4$	Spin coating	85	Maintaining 83% of the original efficiency exposed to 80% R.H. and 50 °C for 600 h (without encapsulation) and 87% under 1 sun illumination for 600 h (encapsulated)	[6]
Glass/FTO/TiO_2/PVK/Polystyrene/Spiro-OMeTAD/Ag	Polystyrene	Spin coating	92.5	Maintaining about 85% initial efficiency after 2 months storage in open air conditions (without encapsulation)	[7]
Glass/FTO/TiO_2/PVK/Pentafluorophenylethylammonium (FEA) lead iodide [$(FEA)_2PbI_4$]/Spiro-OMeTAD/Au/Anti-reflective coating	Pentafluorophenylethyl ammonium (FEA) lead iodide [$(FEA)_2PbI_4$]	Dipping + Spin coating	~ 96	Maintaining 90% of the initial efficiency after 1000 hour at the MPP under 1 sun irradiation in ambient condition with a prevalent humidity of 40% (without encapsulation)	[8]
Glass/ITO/SnO_2/PVK/ PTzNDI-2FT/Spiro-OMeTAD/Au	PTzNDI-2FT interlayer	Spin coating	101.4	Maintaining 96% of the original PCE after being exposed under the condition at 5–10% RH for 3000 h and 97% of the original PCE in a nitrogen-filled glovebox at 85 °C for 500 h (without encapsulation)	[9]
Glass/FTO/TiO_2/PVK/Spiro-OMeTAD/Au/hydrophobic Teflon coating	Hydrophobic Teflon coating	Spin coating	118	Maintaining 95% of the initial PCE after 30 days storage in ambient atmosphere (without encapsulation) and no color changes with a strong stability under water for more than 900 s	[10]
Glass/FTO/NiO/PFDT-modified PVK/PCBM/BCP/ PFDT-modified Ag electrode	PFDT-based self-assembled monolayer	Vapor-assisted self-assembly method	120.1	Maintaining 90.1 % of the initial PCE after 500 h at MPP under 1 Sun (white LED lamp) in a N_2-filled box at 85 °C and retaining nearly the full performance after 500 h aging under RH of 85 ± 10% (without encapsulation) Maintaining the black phase of perovskite layer as long as 45 s immersed under acidic water pH 5.5 (without encapsulation)	[11]

Glass/FTO/SnO_2/TiO_2/PVK/Spiro-OMeTAD/Ag/ Polystyrene (PS)-4033/PS-4033-SiO_2	Polystyrene (PS)-4033/PS-4033-SiO_2	Spray coating	143.4	Maintaining 85 % of the initial PCE after 280 min under humid environment (60–70 % RH) with an ambient temperature of 30–35 °C (without encapsulation)	[12]
Glass/FTO/SnOx/PVK/TFMBA/Spiro-OMeTAD/Au	4-(trifluoromethyl)benzyl amine (TFMBA)	Dynamic spin-coating	106.38°	Unsealed PSCs retaining 84% of the original PCE after aged 1300 h at 65%−75% relative humidity (RH)	[19]
Glass/ITO/PTAA:F_4TCNQ/PVK/TTC/C_{60}/ BCP/Ag	tetratetracontane (TTC, $CH_3(CH_2)_{42}CH_3$)	Thermal evaporation	103°	Maintaining 87% of the initial efficiency after continuous exposure for 200 h in air (40% RH).	[20]
Glass/FTO/TiO_2/ZrO_2/carbon triple layer PSCs/ZrO_2 hydrophobic layer	1H, 1H, 2H, 2H-perfluorooctyltriethoxysilane-based ZrO_2 nanoparticles	Air-brushed coating	**154.5°**	Maintaining superior stability of more than 150 days of outdoor storage, 240 h of continuous operation at the maximum power output point in ambient air with relative humidity as high as ~80%, and stable operation for more than 10 h under raining condition.	[13]
Glass/FTO/TiO_2/PVK/carbon/1H, 1H, 2H, 2H-perfluorodecyltriethoxysilane	1H, 1H, 2H, 2H-perfluorodecyltriethoxy silane	Spraying	**166.4°**	Retaining 99 % of its initial efficiency over 31 days at 95 % relative humidity (RH) and 25-30 °C. The cells (without encapsulation) maintained their PCE in the presence of a UV lamp and retained 98.5% of the initial efficiency at the RH of 90% at 50 °C, over a period of 31 days.	[14]
Glass/ITO/SnO_2/PVK/HTAP spiro-MeOTAD/Au	Hexadecyltrimethylammonium hexafluorophosphate (HTAP)	Spin coating	102.3°	Maintaining 90% of the original PCE after operation at 60 ± 10% RH for 1200 h and exhibited a 20% loss in PCE after 500 h light soaking aging (unencapsulated).	[21]
Glass/FTO/TiO_2/PVK/ Spiro-MeOTAD /carbon/ hydrophobic fumed silica	Hydrophobic fumed silica	Spin coating	**160.08°**	The efficiency loss was about 6.72% of the original value after 90 days of exposure to humidity of averagely over 60 RH% and natural light (unencapsulated).	[22]
Glass/FTO/TiO_2/Al_2O_3/compressed Carbon/graphite nanocomposite (CGn)	Carbon/graphite nanocomposite (CGn)	Compression	140°	Performance of cells are consistent remarkably over 7000 h in ambient atmospheric condition.	[23]

Table S2. Average PV parameters and standard deviation of 17 unencapsulated CPSCs from Batch I before and after super-repellent coating under 1 Sun condition. The change in each average PV parameter is decided by calculating change for each device separately and then mean and standard deviation of the change. P-value is calculated with a two-tailed t-test: paired two sample for means. P-values less than 0.05 (underlined) indicate statistically significant difference. The measured aperture area was 0.64 cm^2.

		J_{SC} (mA/cm^2)	V_{OC} (V)	FF (%)	η (%)
**Forward**	Before	13.0 ± 0.8	0.87 ± 0.01	53 ± 3	6.1 ± 0.3
	After	13.6 ± 0.7	0.86 ± 0.01	51 ± 2	5.9 ± 0.3
	Change (%)	*4.5 ± 6.6*	*-1.3 ± 1.2*	*-4.9 ± 6.4*	*-2.3 ± 2.9*
	p-value	*<u>0.02</u>*	*<u>0.00</u>*	*<u>0.01</u>*	*<u>0.01</u>*
**Reverse**	Before	13.2 ± 0.4	0.90 ± 0.01	73 ± 2	8.7 ± 0.4
	After	13.5 ± 0.6	0.90 ± 0.01	73 ± 3	8.9 ± 0.2
	Change (%)	*2.2 ± 3.6*	*-0.4 ± 0.9*	*1.0 ± 4.0*	*2.8 ± 5.6*
	p-value	*<u>0.02</u>*	*0.11*	*0.36*	*0.08*
**Average**	Before	13.1 ± 0.5	0.89 ± 0.01	62 ± 2	7.2 ± 0.3
	After	13.5 ± 0.6	0.88 ± 0.01	61 ± 2	7.3 ± 0.2
	Change (%)	*3.3 ± 3.7*	*-0.7 ± 0.6*	*-1.0 ± 4.5*	*1.4 ± 4.2*
	p-value	*<u>0.00</u>*	*<u>0.00</u>*	*0.34*	*0.22*

Table S3. Average PV parameters and standard deviation of 8 unencapsulated CPSCs from Batch II before and after super-repellent coating under 1 Sun condition. The change in each average PV parameter is decided by calculating change for each device separately and then mean and standard deviation of the change. P-value is calculated with a two-tailed t-test: paired two sample for means. P-values less than 0.05 (underlined) indicate statistically significant difference. The measured aperture area was 0.64 cm^2.

		J_{SC} (mA/cm^2)	V_{OC} (V)	FF (%)	η (%)
**Forward**	Before	15.7 ± 0.5	0.91 ± 0.01	59 ± 2	8.5 ± 0.2
	After	16.0 ± 0.7	0.90 ± 0.02	57 ± 3	8.1 ± 0.3
	Change (%)	*1.8 ± 2.5*	*-1.0 ± 1.0*	*-4.5 ± 3.6*	*-3.8 ± 2.2*
	p-value	*0.08*	*0.03*	*0.01*	*0.00*
**Reverse**	Before	16.4 ± 0.3	0.91 ± 0.02	68 ± 3	10.1 ± 0.5
	After	16.2 ± 0.3	0.91 ± 0.01	70 ± 2	10.4 ± 0.4
	Change (%)	*-0.8 ± 0.6*	*0.5 ± 0.8*	*3.4 ± 1.4*	*3.1 ± 2.1*
	p-value	*0.01*	*0.11*	*0.00*	*0.00*
**Average**	Before	16.0 ± 0.4	0.91 ± 0.02	63 ± 2	9.2 ± 0.3
	After	16.1 ± 0.5	0.91 ± 0.01	63 ± 3	9.2 ± 0.3
	Change (%)	*0.5 ± 1.2*	*-0.2 ± 0.8*	*-0.7 ± 1.6*	*-0.4 ± 1.2*
	p-value	*0.28*	*0.50*	*0.27*	*0.34*

Table S4. Average (of forward and reverse scans) PV parameters of pristine and super-repellent CPSCs (Batch II) at the initial under 1 Sun and 0.4 Sun and after the water-dropping test under 0.4 Sun. The measured aperture area was 0.64 cm^2.

		J_{SC} (mA/cm^2)	V_{OC} (V)	FF (%)	η (%)	J_{MPP} (mA/cm^2)	V_{MPP} (V)
**Pristine**	Initial 1 Sun	15.9	0.92	68	10.0	14.8	0.68
	Initial 0.4 Sun	6.9	0.90	76	11.9	6.8	0.70
	Final 0.4 Sun	0.6	0.42	27	0.7	0.3	0.22
**Super-repellent**	Initial 1 Sun	16.5	0.92	65	9.7	14.9	0.66
	Initial 0.4 Sun	6.9	0.90	72	11.2	6.7	0.67
	Final 0.4 Sun	7.0	0.88	76	11.7	6.7	0.70

Table S5. Average (of forward and reverse scans) PV parameters and standard deviation of unencapsulated pristine and super-repellent CPSCs (6 cells in each group) under 1 Sun condition in the dark storage aging test at room temperature (RT) and relative humidity (RH) 65% and 85%. The aging test consists of two segments: segment 1 was conducted at RT and 65% RH for 864 hours, and segment 2 was conducted at RT, 85% RH for 165 hours. The difference is the relative difference of average values between pristine and super-repellent groups. P-value is calculated with a two-tailed two-sample t-test with unequal variances. P-values less than 0.05 (underlined) indicate statistically significant difference. The measured aperture area was 0.64 cm^2.

		J_{SC} (mA/cm^2)	V_{OC} (V)	FF (%)	η (%)
**Initial**	Pristine	13.2 ± 0.2	0.89 ± 0.01	63 ± 1	7.4 ± 0.1
	Super-repellent	13.7 ± 0.2	0.88 ± 0.00	60 ± 1	7.3 ± 0.1
	Difference	*3.8 %*	*-0.4 %*	*-4.8 %*	*-1.5 %*
	p-value	*<u>0.00</u>*	*0.21*	*<u>0.00</u>*	*0.14*
**After 65 h of seg. 1**	Pristine	17.0 ± 0.3	0.89 ± 0.01	60 ± 1	9.1 ± 0.2
	Super-repellent	17.2 ± 0.2	0.89 ± 0.01	59 ± 2	9.0 ± 0.3
	Difference	*1.2 %*	*0.0 %*	*-2.2 %*	*-1.0 %*
	p-value	*0.19*	*0.98*	*0.12*	*0.50*
**After seg. 1**	Pristine	16.1 ± 0.2	0.86 ± 0.01	62 ± 1	8.5 ± 0.2
	Super-repellent	16.5 ± 0.2	0.86 ± 0.00	60 ± 1	8.6 ± 0.1
	Difference	*2.5 %*	*0.2 %*	*-1.9 %*	*0.8 %*
	p-value	*<u>0.00</u>*	*0.59*	*<u>0.05</u>*	*0.47*
**After seg. 2**	Pristine	17.4 ± 0.3	0.85 ± 0.00	60 ± 1	8.9 ± 0.2
	Super-repellent	17.8 ± 0.3	0.85 ± 0.00	60 ± 1	9.0 ± 0.3
	Difference	*2.2 %*	*0.1 %*	*-1.4 %*	*0.9 %*
	p-value	*0.06*	*0.63*	*0.12*	*0.56*

Table S6. Average (of forward and reverse scans) PV parameters and standard deviation of unencapsulated pristine and super-repellent CPSCs (6 cells in each group) under 1 Sun condition in the dark storage aging test at room temperature (RT) and relative humidity (RH) 65% and 85%. The aging test consists of two segments: segment 1 was conducted at RT and 65% RH for 864 hours, and segment 2 was conducted at RT, 85% RH for 165 hours. The change in each average PV parameter is decided by calculating change for each device separately (compared to the initial) and then mean and standard deviation of the change. P-value is calculated with a two-tailed t-test: paired two sample for means. P-values less than 0.05 (underlined) indicate statistically significant difference. The measured aperture area was 0.64 cm^2.

		J_{SC} (mA/cm^2)	V_{OC} (V)	FF (%)	η (%)
**Pristine**	Initial	13.2 ± 0.2	0.89 ± 0.01	63 ± 1	7.4 ± 0.1
	After 65 h of seg. 1	17.0 ± 0.3	0.89 ± 0.01	60 ± 1	9.1 ± 0.2
	Change (%)	*29.0 ± 3.3*	*0.5 ± 0.7*	*-5.1 ± 1.7*	*23.0 ± 3.5*
	p-value	*0.00*	*0.14*	*0.00*	*0.00*
	After seg. 1	16.1 ± 0.2	0.86 ± 0.01	62 ± 1	8.5 ± 0.2
	Change (%)	*22.7 ± 2.8*	*-3.2 ± 0.6*	*-2.4 ± 1.4*	*15.9 ± 2.8*
	p-value	*0.00*	*0.00*	*0.01*	*0.00*
	After seg. 2	17.4 ± 0.3	0.85 ± 0.00	60 ± 1	8.9 ± 0.2
	Change (%)	*32.5 ± 3.4*	*-4.2 ± 0.7*	*-4.3 ± 2.1*	*21.5 ± 3.2*
	p-value	*0.00*	*0.00*	*0.00*	*0.00*
**Super-repellent**	Initial	13.7 ± 0.2	0.88 ± 0.00	60 ± 1	7.3 ± 0.1
	After 65 h of seg. 1	17.2 ± 0.2	0.89 ± 0.01	59 ± 2	9.0 ± 0.3
	Change (%)	*25.6 ± 2.2*	*0.9 ± 0.7*	*-2.6 ± 2.5*	*23.5 ± 2.3*
	p-value	*0.00*	*0.03*	*0.06*	*0.00*
	After seg. 1	16.5 ± 0.2	0.86 ± 0.00	60 ± 1	8.6 ± 0.1
	Change (%)	*21.1 ± 1.2*	*-2.7 ± 0.4*	*0.5 ± 1.0*	*18.5 ± 1.7*
	p-value	*0.00*	*0.00*	*0.24*	*0.00*
	After seg. 2	17.8 ± 0.3	0.85 ± 0.00	60 ± 1	9.0 ± 0.3
	Change (%)	*30.4 ± 3.2*	*-3.7 ± 0.7*	*-0.9 ± 1.6*	*24.5 ± 3.4*
	p-value	*0.00*	*0.00*	*0.23*	*0.00*

Table S7. Average change percentage (%) in each PV parameters of unencapsulated pristine and super-repellent CPSCs (6 cells in each group) during the dark storage aging test at room temperature (RT) and relative humidity (RH) 65% and 85% compared to the initial. The aging test consists of two segments: segment 1 was conducted at RT and 65% RH for 864 hours, and segment 2 was conducted at RT, 85% RH for 165 hours. P-value is calculated with two-tailed two-sample t-test with unequal variances for the sample-wise change percentage between the pristine and super-repellent groups. P-values less than 0.05 (underlined) indicate statistically significant difference.

	Avg. change percentage	J_{SC} (%)	V_{OC} (%)	FF (%)	η (%)
**After 65 h of seg. 1**	Pristine	29.0 ± 3.3	0.5 ± 0.7	-5.1 ± 1.7	23.0 ± 3.5
	Super-repellent	25.6 ± 2.2	0.9 ± 0.7	-2.6 ± 2.5	23.5 ± 2.3
	p-value	*0.07*	*0.37*	*0.07*	*0.79*
**After seg. 1**	Pristine	22.7 ± 2.8	-3.2 ± 0.6	-2.4 ± 1.4	15.9 ± 2.8
	Super-repellent	21.1 ± 1.2	-2.7 ± 0.4	0.5 ± 1.0	18.5 ± 1.7
	p-value	*0.24*	*0.10*	<u>*0.00*</u>	*0.09*
**After seg. 2**	Pristine	32.5 ± 3.4	-4.2 ± 0.7	-4.3 ± 2.1	21.5 ± 3.2
	Super-repellent	30.4 ± 3.2	-3.7 ± 0.7	-0.9 ± 1.6	24.5 ± 3.4
	p-value	*0.29*	*0.25*	<u>*0.01*</u>	*0.15*

Table S8. Average (of forward and reverse scans) PV parameters and standard deviation of unencapsulated pristine and super-repellent CPSCs (6 cells in each group) under 1 Sun condition before and after the aging test at elevated temperature (40 °C). The difference is the relative difference of average values between pristine and super-repellent CPSCs. P-value is calculated with a two-tailed two-sample t-test with unequal variances. P-values less than 0.05 (underlined) indicate statistically significant difference. The measured aperture area was 0.64 cm^2.

		J_{SC} (mA/cm^2)	V_{OC} (V)	FF (%)	η (%)
**Before**	Pristine	17.4 ± 0.3	0.85 ± 0.00	60 ± 1	8.9 ± 0.2
	Super-repellent	17.8 ± 0.3	0.85 ± 0.00	60 ± 1	9.0 ± 0.3
	Difference	*2.2 %*	*0.1 %*	*-1.4 %*	*0.9 %*
	p-value	*0.06*	*0.63*	*0.12*	*0.56*
**After 48 h**	Pristine	6.7 ± 1.6	0.82 ± 0.03	63 ± 2	3.4 ± 0.9
	Super-repellent	7.8 ± 2.5	0.81 ± 0.05	63 ± 2	4.1 ± 1.5
	Difference	*16.3 %*	*-0.6 %*	*0.8 %*	*18.2 %*
	p-value	*0.39*	*0.84*	*0.68*	*0.41*

Table S9. Average (of forward and reverse scans) PV parameters and standard deviation of unencapsulated pristine and super-repellent CPSCs (6 cells in each group) under 1 Sun condition before and after the aging test at elevated temperature (40 °C). The change in each average PV parameter is decided by calculating change for each device separately (compared to the before) and then mean and standard deviation of the change. P-value is calculated with two-tailed t-test: paired two sample for means. P-values less than 0.05 (underlined) indicate statistically significant difference. The measured aperture area was 0.64 cm^2.

		J_{SC} (mA/cm^2)	V_{OC} (V)	FF (%)	η (%)
**Pristine**	Before	17.4 ± 0.3	0.85 ± 0.00	60 ± 1	8.9 ± 0.2
	After 48 h	6.7 ± 1.6	0.82 ± 0.03	63 ± 2	3.4 ± 0.9
	Change (%)	*-61.6 ± 8.8*	*-4.0 ± 3.7*	*3.6 ± 2.6*	*-61.7 ± 10.0*
	p-value	*0.00*	*0.05*	*0.02*	*0.00*
**Super-repellent**	Before	17.8 ± 0.3	0.85 ± 0.00	60 ± 1	9.0 ± 0.3
	After 48 h	7.8 ± 2.5	0.81 ± 0.05	63 ± 2	4.1 ± 1.5
	Change (%)	*-56.4 ± 13.6*	*-4.8 ± 5.9*	*5.9 ± 3.3*	*-55.2 ± 15.9*
	p-value	*0.00*	*0.1*	*0.01*	*0.00*

Table S10. Average change percentage (%) in each PV parameter of unencapsulated pristine and super-repellent CPSCs (6 cells in each group) after the aging test at elevated temperature (40 °C) compared to the before. P-value is calculated with two-tailed two-sample t-test with unequal variances for the sample-wise change percentage between the pristine and super-repellent groups. P-values less than 0.05 (underlined) indicate statistically significant difference.

Avg. change percentage	J_{SC} (%)	V_{OC} (%)	FF (%)	η (%)
Pristine	-61.6 ± 8.8	-4.0 ± 3.7	3.6 ± 2.6	-61.7 ± 10.0
Super-repellent	-56.4 ± 13.6	-4.8 ± 5.9	5.9 ± 3.3	-55.2 ± 15.9
p-value	*0.45*	*0.79*	*0.21*	*0.43*

Table S11. Average (of forward and reverse scans) PV parameters and standard deviation of encapsulated pristine and super-repellent CPSCs (6 cells in each group) under 1 Sun condition at the initial and after the dark storage test at 65 °C in ambient. The difference is the relative difference of average values between pristine and super-repellent CPSCs. P-value is calculated with a two-tailed two-sample t-test with unequal variances. P-values less than 0.05 (underlined) indicate statistically significant difference. The measured aperture area was 0.64 cm^2.

		J_{SC} (mA/cm^2)	V_{OC} (V)	FF (%)	η (%)
**Initial**	Pristine	13.4 ± 0.4	0.89 ± 0.00	63 ± 1	7.5 ± 0.2
	Super-repellent	13.5 ± 0.5	0.89 ± 0.01	63 ± 1	7.6 ± 0.2
	Difference	*0.5 %*	*0.8 %*	*0.1 %*	*1.3 %*
	p-value	*0.82*	*0.09*	*0.96*	*0.47*
**After 981 h**	Pristine	10.9 ± 0.6	0.77 ± 0.02	58 ± 2	4.9 ± 0.3
	Super-repellent	11.5 ± 0.7	0.78 ± 0.03	57 ± 2	5.0 ± 0.3
	Difference	*5.3 %*	*1.2 %*	*-2.8 %*	*3.5 %*
	p-value	*0.16*	*0.48*	*0.25*	*0.31*

Table S12. Average (of forward and reverse scans) PV parameters and standard deviation of encapsulated pristine and super-repellent CPSCs (6 cells in each group) under 1 Sun condition at the initial and after the dark storage test at 65 °C in ambient. The change in each average PV parameter is decided by calculating change for each device separately (compare to the initial) and then mean and standard deviation of the change. P-value is calculated with a two-tailed t-test: paired two sample for means. P-values less than 0.05 (underlined) indicate statistically significant difference. The measured aperture area was 0.64 cm^2.

		J_{SC} (mA/cm^2)	V_{OC} (V)	FF (%)	η (%)
**Pristine**	Initial	13.4 ± 0.4	0.89 ± 0.00	63 ± 1	7.5 ± 0.2
	After 981 h	10.9 ± 0.6	0.77 ± 0.02	58 ± 2	4.9 ± 0.3
	Change (%)	*-18.9 ± 4.7*	*-13.3 ± 1.7*	*-7.8 ± 3.5*	*-35.3 ± 2.2*
	p-value	*0.00*	*0.00*	*0.00*	*0.00*
**Super-repellent**	Initial	13.5 ± 0.5	0.89 ± 0.01	63 ± 1	7.6 ± 0.2
	After 981 h	11.5 ± 0.7	0.78 ± 0.03	57 ± 2	5.0 ± 0.3
	Change (%)	*-15.1 ± 3.2*	*-12.9 ± 3.5*	*-10.4 ± 3.2*	*-33.8 ± 2.9*
	p-value	*0.00*	*0.00*	*0.00*	*0.00*

Table S13. Average change percentage (%) in each PV parameters of encapsulated pristine and super-repellent CPSCs (6 cells in each group) after the dark storage test at 65 °C in ambient compared to the before. P-value is calculated with a two-tailed two-sample t-test with unequal variances for the sample-wise change percentage between the with and without SHC groups. P-values less than 0.05 (underlined) indicate statistically significant difference.

Avg. change percentage	J_{SC} (%)	V_{OC} (%)	FF (%)	η (%)
Pristine	-18.9 ± 4.7	-13.3 ± 1.7	-7.8 ± 3.5	-35.3 ± 2.2
Super-repellent	-15.1 ± 3.2	-12.9 ± 3.5	-10.4 ± 3.2	-33.8 ± 2.9
p-value	*0.13*	*0.82*	*0.20*	*0.36*

Table S14. Average (of forward and reverse scans) PV parameters and standard deviation of unencapsulated pristine and super-repellent CPSCs (6 cells in each group) under 1 Sun condition at the initial and after condensation test. The difference is the relative difference of average values between pristine and super-repellent CPSCs. The p-value is calculated with a two-tailed two-sample t-test with unequal variances. P-values less than 0.05 indicate a statistically significant difference. The measured aperture area was 0.64 cm^2.

		J_{SC} (mA/cm^2)	V_{OC} (V)	FF (%)	η (%)
**Initial**	Pristine	16.3 ± 0.6	0.91 ± 0.00	61 ± 1	9.1 ± 0.5
	Super-repellent	16.3 ± 0.3	0.90 ± 0.01	62 ± 1	9.0 ± 0.1
	Difference	*0.1 %*	*-1.1 %*	*0.3 %*	*-0.7 %*
	p-value	*0.96*	*0.10*	*0.69*	*0.74*
**After**	Pristine	1.1 ± 1.3	0.76 ± 0.04	44 ± 4	0.4 ± 0.5
	Super-repellent	0.2 ± 0.2	0.72 ± 0.05	39 ± 4	0.1 ± 0.1
	Difference	*-79.4 %*	*-4.7 %*	*-11 %*	*-83.7 %*
	p-value	*0.16*	*0.19*	*0.07*	*0.17*

Description of Supplementary Videos.

Supplementary Video S1. Pristine CPSC undergoes degradation upon exposure to macroscopic droplets (~8 µL).

Supplementary Video S2. Immobile droplet on one-layer coated weak-repellent CPSC.

Supplementary Video S3. Mobile droplet on three-layer coated super-repellent CPSC.

Supplementary Video S4. Bouncing droplet on super-repellent CPSC.

Supplementary Video S5. Rain falling experiment for pristine CPSC.

Supplementary Video S6. Rain falling experiment for super-repellent CPSC.

Supplementary Video S7. Bouncing droplets on super-repellent CPSC during rain falling experiment.

Supplementary Video S8. Super-repellent coating after condensation experiments.

References

[1] Y. Shi, F. Zhang, *Solar RRL* **2023**, *7*, 2201123.
[2] A. Marmur, C. Della Volpe, S. Siboni, A. Amirfazli, J. W. Drelich, *Surface Innovations* **2017**, *5*, 3.
[3] D. Daniel, M. Vuckovac, M. Backholm, M. Latikka, R. Karyappa, X. Q. Koh, J. V. I. Timonen, N. Tomczak, R. H. A. Ras, *Communications Physics* **2023**, *6*, 152.
[4] K. Liu, M. Vuckovac, M. Latikka, T. Huhtamäki, R. H. A. Ras, *Science* **2019**, *363*, 1147.
[5] D. Daniel, M. Vuckovac, M. Backholm, M. Latikka, R. Karyappa, X. Q. Koh, J. V. I. Timonen, N. Tomczak, R. H. A. Ras, *Commun Phys* **2023**, *6*, 152.
[6] W. Shi, H. Ye, *The Journal of Physical Chemistry Letters* **2021**, *12*, 4052.
[7] M. Li, X. Yan, Z. Kang, Y. Huan, Y. Li, R. Zhang, Y. Zhang, *ACS Applied Materials & Interfaces* **2018**, *10*, 18787.
[8] L. Yuhang, A. Seckin, P. Linfeng, U. Ryusuke, A. Neha, M. J. V., H. Alexander, S. Frank, U. A. R., Z. S. M., H. Anders, D. M. Ibrahim, G. Michael, *Science Advances* **2022**, *5*, eaaw2543.
[9] Y. Li, E. L. Lim, H. Xie, J. Song, T. Kong, Y. Zhang, M. Yang, B. Wu, C. Duan, D. Bi, *ACS Photonics* **2021**, *8*, 3185.
[10] I. Hwang, I. Jeong, J. Lee, M. J. Ko, K. Yong, *ACS Applied Materials & Interfaces* **2015**, *7*, 17330.
[11] H. Zhang, K. Li, M. Sun, F. Wang, H. Wang, A. K.-Y. Jen, *Advanced Energy Materials* **2021**, *11*, 2102281.
[12] Z. Luo, C. Zhang, L. Yang, J. Zhang, *ChemSusChem* **2022**, *15*, e202102008.
[13] J. Luo, H. Bin Yang, M. Zhuang, S. Liu, L. Wang, B. Liu, *Journal of Energy Chemistry* **2020**, *50*, 332.
[14] R. Keshavarzi, N. Molabahrami, N. Afzali, M. Omrani, *Solar RRL* **2020**, *4*, 2000491.
[15] J. V. I. Timonen, M. Latikka, O. Ikkala, R. H. A. Ras, *Nature Communications* **2013**, *4*, 2398.
[16] M. Junaid, H. A. Nurmi, M. Latikka, M. Vuckovac, R. H. A. Ras, *Droplet* **2022**, *1*, 38.
[17] A. Ricchiuto, A. Tozzi, *American Journal of Physics* **1982**, *50*, 176.
[18] A. I. ElSherbini, A. M. Jacobi, *Journal of colloid and interface science* **2006**, *299*, 841.
[19] J. Li, T. Bu, Z. Lin, Y. Mo, N. Chai, X. Gao, M. Ji, X.-L. Zhang, Y.-B. Cheng, F. Huang, *Chemical Engineering Journal* **2021**, *405*, 126712.
[20] Z. Li, J. Dong, C. Liu, J. Guo, L. Shen, W. Guo, *Nano-Micro Letters* **2019**, *11*, 50.
[21] S. Xu, L. Zhang, B. Liu, Z. Liang, H. Xu, H. Zhang, J. Ye, H. Ma, G. Liu, X. Pan, *Chemical Engineering Journal* **2023**, *453*, 139808.
[22] S. Abbasi, X. Wang, P. Tipparak, C. Bhoomanee, P. Ruankham, H. Liu, D. Wongratanaphisan, W. Shen, *Materials Science in Semiconductor Processing* **2023**, *155*, 107241.
[23] S. K. Yadav, *Materials Chemistry and Physics* **2021**, *268*, 124709.
